# Frequency of malaria and glucose-6-phosphate dehydrogenase deficiency in Tajikistan

**DOI:** 10.1186/1475-2875-5-51

**Published:** 2006-06-16

**Authors:** Cornelia E Rebholz, Anette J Michel, Daniel A Maselli, Karimov Saipphudin, Kaspar Wyss

**Affiliations:** 1 Swiss Centre for International Health, Swiss Tropical Institute, P.O.-Box, Socinstrasse 57, CH-4002 Basel, Switzerland; 2 Centre for Development and Environment, Institute of Geography, University of Berne, Steigerhubelstrasse 3, CH-3008 Berne, Switzerland; 3 Republican Centre of Tropical Diseases, Ministry of Health, Alischera Nawon street 5/4, Dushanbe, Tajikistan

## Abstract

**Background:**

During the Soviet era, malaria was close to eradication in Tajikistan. Since the early 1990s, the disease has been on the rise and has become endemic in large areas of southern and western Tajikistan. The standard national treatment for *Plasmodium vivax *is based on primaquine. This entails the risk of severe haemolysis for patients with glucose-6-phosphate dehydrogenase (G6PD) deficiency. Seasonal and geographical distribution patterns as well as G6PD deficiency frequency were analysed with a view to improve understanding of the current malaria situation in Tajikistan.

**Methods:**

Spatial and seasonal distribution was analysed, applying a risk model that included key environmental factors such as temperature and the availability of mosquito breeding sites. The frequency of G6PD deficiency was studied at the health service level, including a cross-sectional sample of 382 adult men.

**Results:**

Analysis revealed high rates of malaria transmission in most districts of the southern province of Khatlon, as well as in some zones in the northern province of Sughd. Three categories of risk areas were identified: (i) zones at relatively high malaria risk with high current incidence rates, where malaria control and prevention measures should be taken at all stages of the transmission cycle; (ii) zones at relatively high malaria risk with low current incidence rates, where malaria prevention measures are recommended; and (iii) zones at intermediate or low malaria risk with low current incidence rates where no particular measures appear necessary. The average prevalence of G6PD deficiency was 2.1% with apparent differences between ethnic groups and geographical regions.

**Conclusion:**

The study clearly indicates that malaria is a serious health issue in specific regions of Tajikistan. Transmission is mainly determined by temperature. Consequently, locations at lower altitude are more malaria-prone. G6PD deficiency frequency is too moderate to require fundamental changes in standard national treatment of cases of *P. vivax*.

## Background

Under Soviet rule, malaria was reported having been nearly eradicated in Tajikistan by the end of the 1960s; only in the region bordering Afghanistan, in the southern part of the country, did a low level of *Plasmodium vivax *transmission persist [1]. However, since 1992 the situation appears to have deteriorated considerably. The World Health Organization (WHO) estimates annual incidences to be much higher than what is officially reported. Estimates are in the range of 300–400,000 cases of *P. vivax *and 30–50,000 cases of *Plasmodium falciparum *[2]. This contrasts with official governmental statistics indicating a peak of 30,000 cases in 1997, followed by a decline [3]. According to the same governmental data, more than 80% of the cases were registered in the southern province of Khatlon, where there is a substantial influx of Tajik refugees returning after the civil war from northern Afghanistan. In 1997, many Afghans also entered Tajikistan from the south, fleeing the Taliban regime and, thus, possibly importing malaria parasites. With the deterioration of basic infrastructure and social services due to the civil war, all malaria prevention measures were suspended and drainage systems are no longer adequately maintained. These developments provide a better breeding ground for mosquitoes in ditch-water reservoirs and other stagnant pools [2].

Since the early 1990s, changes in spatial malaria distribution patterns have been observed. An increase in average temperatures and changes in land use are seen as driving factors here [1]. In areas where the climate is marginally suitable for malaria transmission – such as areas at high altitude in Tajikistan – climatic variations may play a key role in the incidence of malaria [4].

The current standard national malaria treatment of *P. vivax *malaria consists in administrating a three-day course of chloroquine followed by a 14-day course of primaquine [5]. Primaquine is the only available antimalarial drug able to effectively eliminate *P. vivax *by killing the latent liver stages of the parasite. This treatment also plays an essential role in controlling the disease by preventing further transmission. However, there is a potential lack of compliance with the treatment due to haemolytic side effects of primaquine. People with an inherited G6PD deficiency are particularly at risk of severe haemolysis when using primaquine. Therefore, India and Pakistan have adhered to a five-day course instead of the recommended 14-day course as their standard malaria treatment. This makes it possible to limit incidences of severe haemolysis, especially in countries where the necessary facilities for detecting G6PD deficiency are often lacking. However, this strategy has the disadvantage of failing to effectively prevent relapses [6]. The general frequency of G6PD varies among areas and ethnic groups [7] and its geographic distribution has led several authors to suggest that G6PD deficiency is a polymorphism that builds resistance to *P. falciparum *malaria [8]. In Tajikistan, a G6PD deficiency frequency of 1.6% in the South and 0% in the North of the country has been investigated by Russian scientists in the 1980ies [9]. Before mutation analysis was possible three different variants of G6PD gene mutations were detected: Dushanbe I and II, which have less than 10% of residual enzyme activity and Dushanbe III with normal enzyme activity [10]. More recent G6PD deficiency studies in Central Asia have revealed quite a range in the frequency of the trait among the different ethnic groups: 2.1% among Irani to 2.9% among Afghan Tajiks over 15.8% among Pakistani Pathans up to 36.4% among Azerbaijani [11-13].

So far, much remains unknown about the current malaria situation in Tajikistan. The present study aims (i) to establish a malaria risk map with special consideration of seasonal and environmental distribution patterns; (ii) to assess G6PD deficiency prevalence and (iii) to critically review the national strategy for combating *P. falciparum *malaria. Jointly, these aims should make it possible to provide relevant information to successfully control the disease in Tajikistan.

## Methods

### Malaria risk model

**Figure 1:**
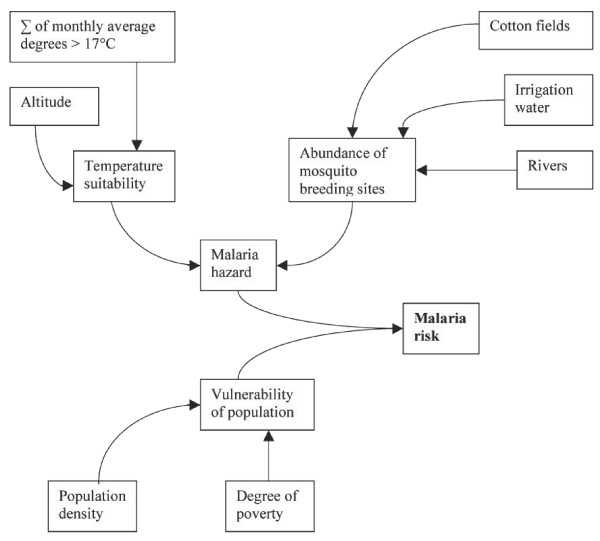
**Simplified malaria risk model**. The model combines the seven mapped datasets to estimate the risk of malaria in Tajikistan.

Figure 1 presents the model that was developed to map spatial distribution of malaria risk in Tajikistan. The influence of plausible key environmental factors on malaria transmission was estimated by analysing available data on climate, agriculture and malaria incidence. It should be mentioned that, as in other places in Central Asia, the general scarcity, discontinuity and questionable reliability of both official and unofficial data remain a challenge. Altogether, seven datasets were used – altitude, sum of monthly average degrees >17°C, area of irrigated cotton fields, length of rivers, amount of irrigation water, population density, and degree of poverty. To complement the findings, field observations and qualitative interviews with local health staff were conducted. To obtain risk exposure to the disease, the model combines the estimated suitability of environmental conditions for malaria transmission – the so-called 'hazard' – with the expected vulnerability of the population to malaria for all of Tajikistan: Risk = Hazard * Vulnerability.

The hazard is characterized and determined by two key factors: temperature and presence of aquatic breeding sites. The suitability of temperature in a specific location is determined (a) by the sum of average monthly temperature above the threshold of 17°C for the onset of malaria transmission, which indicates the degree of temperature suitability as well as the duration of the suitable period; and (b) by altitude above sea level. For (a), the dataset based on the 0.5° grid of the International Institute of Applied Systems Analyses (IIASA) was used. The extent of aquatic breeding sites was estimated using three proxy measures: (i) area cultivated for cotton in ha, (ii) amount of water used for irrigation in m^3^/m^2 ^per year, and (iii) river length in km. For (i) and (ii), data were obtained from the Department of State Statistics and the Ministry of Water Resources and Melioration, respectively. Total river length per district was calculated applying ArcView on the Vector Map Level 0 database provided by the National Imagery and Mapping Agency in the USA. This made it possible to extract 'larger perennial rivers' and 'smaller rivers and streams' by applying the "LengthCoordTool" extension. All three indicators were obtained at district level and then normalized, considering the area of the districts as well as the maximum value. The vulnerability of the population was assessed by combining population density provided by the Department of State Statistics with the poverty coefficient. The latter was given three values based on the Tajik Living Standard Survey conducted by World Bank, which distinguishes 'poor', 'very poor', and 'extremely poor' districts [14].

### G6PD deficiency prevalence

#### Study sites

The study on G6PD deficiency was carried out in three districts: the capital of Dushanbe and two rural districts in the south of Tajikistan, Dangara and Kubodiyon. While Dushanbe has around 560,000 inhabitants and is the largest city in Tajikistan, Dangara and Kubodiyon districts each host a population of around 100,000 [15]. The population of Dangara is assumed to be homogenous, while the district of Kubodiyon, which borders Afghanistan and is close to Uzbekistan, has a more diverse ethnic composition. Dushanbe also hosts most of the ethnic groups living in the Republic.

In Dangara and Kubodiyon official malaria incidence rates in 2003 were quite high, with 210 and 146 cases per 100,000 inhabitants respectively. Dushanbe had a relatively low malaria incidence rate of 38 per 100,000 inhabitants [16].

#### Study design

Since G6PD deficiency is an X-linked trait, only men between the ages of 18 and 50 years were included in the survey. The study participants were recruited at seven of the nine public polyclinics in the city of Dushanbe, at the Central District Hospital in Dangara, and the two major polyclinics in the district in Kubodiyon. On a given day all consulting male outpatients and visitors meeting the study criteria were asked to visit the laboratory room for a blood sample. In addition to laboratory examination, participants were asked to fill in a short structured questionnaire. Prior to sampling, the Ministry of Health had been asked for official permission for the survey. Subsequently, informed written consent was obtained from all study participants.

200 μl of capillary blood was taken from each person using "accucheck softclicks" (F. Hoffmann-La Roche Ltd. Switzerland) and ethylenediaminetetraacetic acid tubes (Aichele Medico AG Switzerland). The haemoglobin content was measured with a portable haemoglobinometer "haemocue+" (HemoCue AG Switzerland). Corresponding to the haemoglobin concentration, a haemolysate with distilled water was prepared and a qualitative G6PD screening test by dye reduction method (Lab care diagnostics Pvt. Ltd. India) was carried out. In this test, the rate of decolourization from blue to the original red colour of the haemolysate corresponds proportionally to enzyme activity. The vials were stored for one hour in a thermostad if one was available in working condition, or put in the sun, stored inside a cardboard box and controlled with a thermometer. Samples that had not changed colour after one hour were recorded as deficient.

## Results

### Spatial and seasonal distribution of malaria

**Figure 2:**
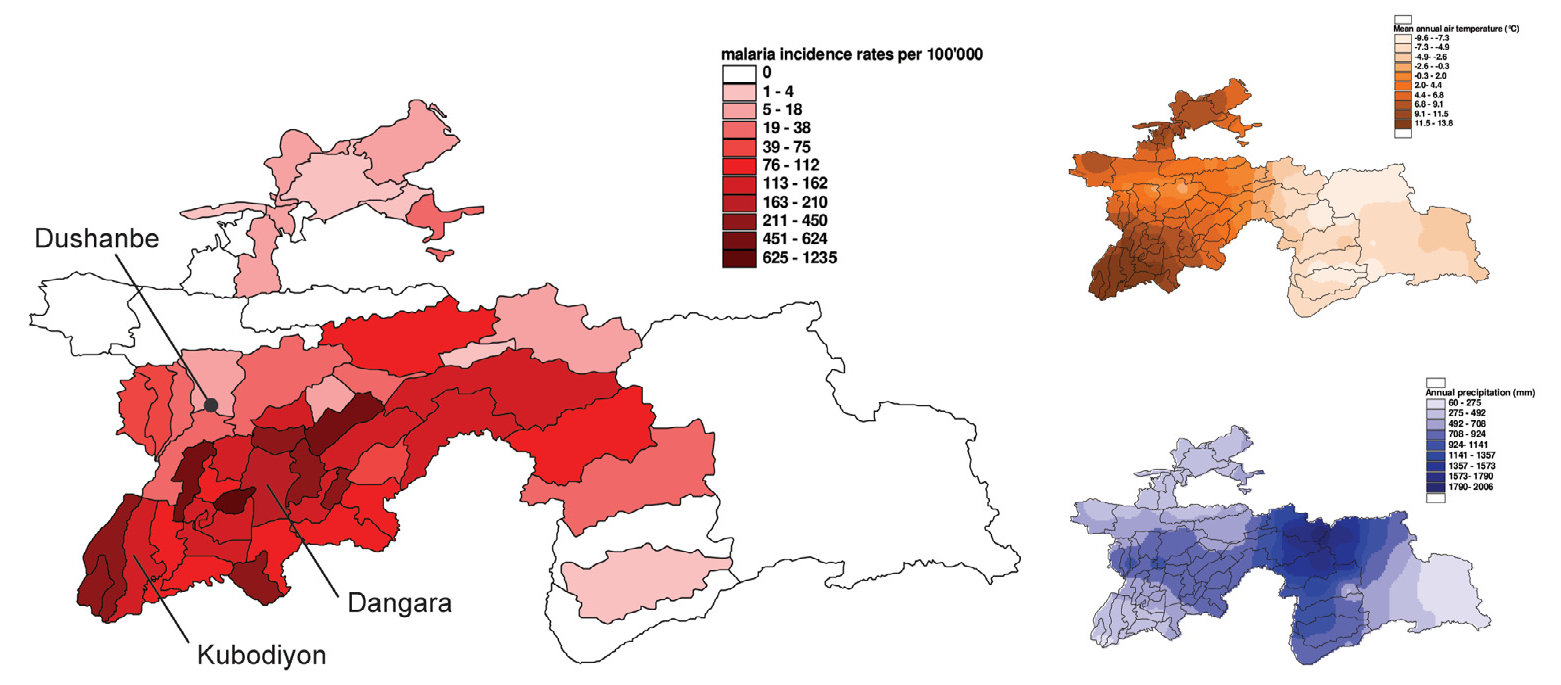
**Malaria distribution versus annual temperature and rainfall patterns**. The incidence of malaria in 2003 in the districts matches annual temperature patterns, but does not coincide with annual rainfall patterns in Tajikistan.

A positive linear correlation was found between the annual long-term average temperature and the annual malaria incidence in 2003 for the eight districts in which a full dataset was available (R^2 ^= 0.27, n = 8) (Figure 2). The correlation showed a narrow range of malaria incidence rates at low temperatures, and a broad range at higher annual average temperatures. Malaria transmission of *P. vivax *is a seasonal phenomenon in Tajikistan. An R^2^-value of 0.58 resulted in the linear regression of monthly long-term average temperature and the percentage of malaria cases per month in 2003 for eight districts in Tajikistan (correlation coefficient = 0.77, n = 96). Incidences usually are reported from April or early May onwards, with a peak in August and a reduction to low levels in October. The *P. falciparum *transmission season begins later, at the end of June and the beginning of July, with a peak in October and a slower decrease than for *P. vivax*, reaching low levels after mid December. The correlation of mean malaria incidence rates during the summer months of June, July and August 2003 with the sum of rainfall during the same period for eight districts was mainly negative. No correlation between cotton cultivation or irrigation and malaria incidence could be observed for Tajikistan in the present study. However, additional qualitative interviews with trained health staff emphasised such possible links.

**Figure 3:**
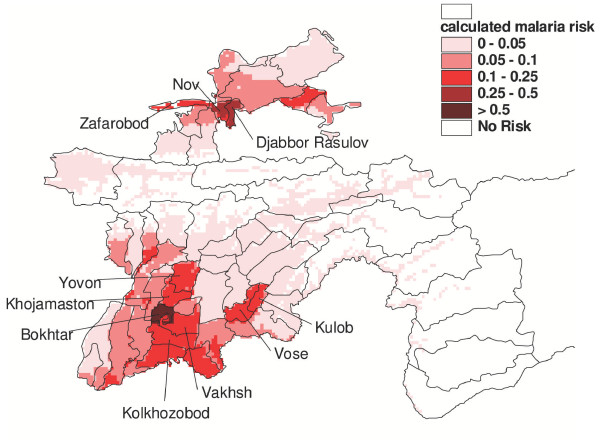
**Malaria risk map**. The malaria risk map shows where high incidence rates can be expected, should the disease occur there as a result of local environmental suitability and vulnerability of the population to transmission.

The malaria risk map obtained shows that the zones at greatest risk are located in the southern and northern parts of the country (Figure 3). This confirms that higher altitudes are free of the risk of malaria. In most districts of the southern province of Khatlon, conditions are very conducive to malaria transmission, due to appropriate temperatures during a relatively long period in summer. An abundance of aquatic breeding places for *Anopheles *is reported for large parts of this region, since cotton cultivation and intense irrigation are widespread. While temperature suitability follows a gradient from south-west to north-east in the province, breeding-places are distributed heterogeneously. In addition, the majority of the districts with highly vulnerable populations are located in Khatlon. The district for which the model clearly indicates the highest risk has, beside appropriate temperatures, the highest proportion of cotton fields per area (over 31%), the highest amount of water used for irrigation per area (> 1.1 m^3^/m^2 ^per year), and a highly vulnerable population, with a population density of 390 people per square kilometre. In the northern province of Tajikistan, high-risk malaria zones are limited to some areas in the centre of the province, as it is mainly in these areas that the calculated temperature index reaches a high level of suitability. The estimated mosquito breeding site availability is high in the entire northern part of the province. The vulnerability values of districts in this province are comparable to districts in Khatlon and result mainly from high population density. Poverty in this region of Tajikistan is generally lower than in the southern and eastern parts of the country.

### Prevalence of G6PD deficiency

Young men 18 to 30 years of age constituted the majority of the study participants in all three districts (≥ 44%). Their proportion was higher in the capital (57%) than in rural districts (45% and 44% respectively). This reflects the proportion of young men in the male population in Tajikistan. In the district that borders Afghanistan, the majority of the study participants (58%) were of Uzbek origin, whereas Tajik men dominated (>89%) at the two other study sites.

**Figure 4:**
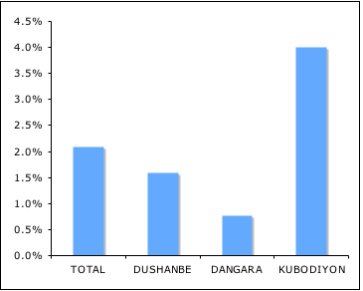
**Prevalence of G6PD deficiency**. Average national G6PD deficiency prevalence and varibility among affected districts in southwest Tajikistan.

An overall G6PD deficiency prevalence of 2.1% was found (8 out of 382). The prevalence varied across the three selected districts from 0.8% (1 out of 131) in Dangara to 1.6% (2 out of 126) in Dushanbe and 4% (5 out of 125) in Kubodiyon (Figure 4). Thereby, the frequency of G6PD deficiency did not match with the reported malaria incidence rates in 2003 of these districts. On the contrary, the district with the highest malaria incidence rate showed the lowest G6PD prevalence rate. But a tendency among participants with Uzbek ethnic roots to have a higher risk of inheritance of G6PD deficiency than participants with Tajik ethnic origin was observed, even though the Chi-squared test was not statistically significant (Table 1). Four out of 93 Uzbek- and four out of 260 Tajik-speaking participants were found to be G6PD deficient. In addition, this trend was supported by statistically significant Uzbek study participants reporting more frequently haemolytic episodes among relatives after the application of primaquine in the district of Dangara (X-squared = 4.97, p-value = 0.026). In our study other potential risk factors for G6PD deficiency, such as socio-economic status and migration, did not yield meaningful statistical results since the number of G6PD-deficient study participants was too small.

**Table 1:** G6PD deficiency and "risk factors". "Risk factors" for G6PD deficiency such as ethnic origin, socio-economic status, migration, and malaria infections, assessed in the questionnaire.

**Risk factors**	DUSHANBE (deficient/total)	DANGARA (deficient/total)	KUBODYION (deficient/total)	TOTAL (deficient/total)
**Ethnic origin**				
Tajik	2/109	1/115	1/53	4/277
Uzbek	0/9	0/14	4/71	4/94
Tajiks reporting haemolysis among relatives	4/109	6/115	3/53	13/27
Uzbeks reporting haemolysis among relatives	0/9	4/13*	2/71	6/93
**Socio-economic status**				
Class I	0/25	0/34	0/25	0/84
Class II	1/14	0/21	1/24	2/59
Class III	0/8	1/26	2/26	3/60
Class IV	0/28	0/30	2/40	2/98
Class V	1/50	0/19	0/10	1/79
**Migration**				
Working abroad	1/15	0/2	0/9	1/26
Never worked abroad	1/104	1/128	5/116	7/348
Originating from the district	1/36	1/121	5/119	7/276
**Malaria infections**				
Previous malaria infection	0/9	0/20	1/47	1/76
Never suffered from malaria before	2/116	1/109	4/77	7/302

* significant (X-squared = 4.9704, p-value = 0.02578)

## Discussion

### Spatial and seasonal distribution of malaria

Low temperatures limit malaria transmission both spatially and temporally. Due to low temperatures, locations at high altitudes are spared the risk of malaria. The threshold for malaria transmission was estimated to be at altitudes higher than 2,500 m above sea level. Malaria epidemics at relatively high altitudes appear to be possible in Central Asia, as one study reported an outbreak in neighbouring Afghanistan at an altitude of around 2,400 m [17]. This excludes the entire high-mountain area in the eastern part of the country – mainly the Pamirs – from malaria transmission.

The dominant role of temperature in transmission is obvious, given the fact that rainfall is scarce if not completely absent during summer in many parts of Tajikistan. The amount of rainfall does thus not appear to influence malaria distribution significantly (Figure 2). In Pakistan, where similar seasonal patterns in *P. vivax *and *P. falciparum *transmission have been observed [18], the malaria transmission season generally follows the monsoon rains [19]. Despite the absence of rainfall in summer, when temperatures are appropriate for malaria vectors to breed, there are plenty of open water bodies due to the high level of water discharge from snow and ice melting in the mountains. Especially irrigation systems in the plains and valleys, but also natural rivers and possibly private water collectors, provide appropriate aquatic habitats for malaria vectors. A study conducted in South Punjab (Pakistan), where rainfall is also low and irrigation is also practised on a large scale, showed most breeding sites were linked to the irrigation system [20].

The main cause of higher malaria incidences in southern Tajikistan compared to northern regions of the country, despite similar suitable environmental conditions, is probably the massive influx of migrants in the mid 1990s from malaria-endemic areas in neighbouring Afghanistan. Apparently the different degrees of poverty in these two parts of the country only play a minor role. Degrees of poverty are known to be less severe in the northern than in the southern province of Tajikistan. Today internal migration is seen as more important for malaria transmission than cross-border movements. However, further investigation is needed to understand the relation between internal and external migration, other socio-economic factors, and malaria transmission patterns in Central Asia.

### Validation of the model

Even though the model used was relatively simple, it appears to be highly plausible with respect to better understanding of the mechanisms of malaria transmission in Tajikistan. While the relative importance of the different factors influencing malaria distribution – such as the degree of immunity in the population, the quality of health services, and preventive measures – is not fully known, the model includes the most important environmental factors that directly influence the biology of the malaria parasites and vectors: temperature and potential aquatic breeding sites.

### Malaria risk zones

As a result of the malaria risk map, three main categories of malaria risk in Tajikistan were identified. They can be characterized as follows:

• Zones at high relative risk of malaria combined with high current incidence rates. This was mainly true for the province of Khatlon in southern Tajikistan. In this zone malaria control and prevention measures should be taken at all stages of the transmission cycle.

• Zones at relatively high risk of malaria but with low current incidence rates. This mainly applies to the northern part of the country. Here the incidence of malaria should be monitored carefully, since some areas located in the centre appear to be at relatively high risk as well.

• Zones at moderate or low risk combined with low current incidence rates. This applies mainly to locations at intermediate altitudes where temperature appears to limit the level of malaria transmission. Accordingly, a possible rise in temperature, which has been prognosticated for the long run due to climate change [21], could lead to an extension and intensification of malaria transmission in these areas. Thus, rising average temperatures would allow malaria to spread to so far malaria-free areas, which then would become especially prone to epidemics due to the lack of immunity among the population [4].

### Prevalence of G6PD deficiency

#### Reliability of the test

The qualitative screening test applied for G6PD deficiency using the dye reduction method worked well. The simplified incubation method apparently did not influence the results, since the decolourization of the test was obvious and the results could be easily determined. The specificity of the test was estimated to be high, as only four participants theoretically could have been deficient while showing a negative result for G6PD deficiency. According to the greater activity of G6PD in young red blood cells, a high amount of reticulocytes in blood can give a false negative result. One study participant was suffering from anaemia, two had liver problems, and one was suspected of having malaria.

#### Regional differences in the frequency of G6PD deficiency

The revealed regional differences in the frequency of G6PD deficiency appear to be related to the ethnic composition of the study populations rather than to malaria incidence rates or other potential determinants. Indeed, the results of this research confirmed previous results obtained in a survey about G6PD deficiency among refugees in Northern Pakistan: a high rate of G6PD deficiency of 9.1% among Uzbek refugees, and a rate of only 2.9% among Tajik refugees [12]. The higher frequency of G6PD deficiency among Uzbeks might be explained by their residence in *P. falciparum *endemic areas in the northern provinces of Afghanistan for many centuries. Information gathered from Russian explorers at the beginning of the 20th century indicates that in southern Uzbekistan and Western Kuhistan, which includes the area of the province of Khatlon, present-day Uzbeks comprised a large number of small tribes, each with specific locations and tribal affiliations. Qunqurat tribes settled in northern Afghanistan during the 14th and 15th centuries. At the end of the 19th and the beginning of the 20th centuries, they were still semi-nomadic, and can be found today in Southern Tajikistan [22]. An effect of selection through *P. falciparum *can therefore not be excluded. In Tajikistan, it is estimated that the *P. falciparum *burden was not great enough to exert selective pressure.

## Conclusion

Malaria transmission in Tajikistan is a mono-seasonal phenomenon, lasting mainly from April to November. Temperature appears to be the main determining environmental factor, not only for seasonal but also for spatial distribution patterns. Based on the established risk map, three different risk zones could be identified:

(i) zones with a high relative risk of malaria combined with high current incidence rates, which can be found in districts of the southern province of Khatlon. In these zones it is recommended to take preventive measures at all stages of the malaria transmission cycle.

(ii) zones at relatively high risk of malaria combined with low current incidence. Here it is recommended to carefully observe the malaria situation and be ready to take measures if necessary.

(iii) zones with intermediate and low risk of malaria combined with low current incidence rates. This is true for locations at intermediate altitudes. A rise in temperature can lead to an increase in malaria incidence and would, moreover, allow malaria transmisson to spread to areas so far free from malaria. Such new areas would be particularly prone to malaria epidemics.

The 2.1% of G6PD deficiency prevalence found suggests that current national standard malaria treatment guidelines for *P. vivax *should be maintained. However, in order to limit incidences of severe haemolysis after primaquine treatment, family members of patients who have had haemolytic episodes should be screened preventively for G6PD deficiency. Persons who have been tested positive should be given a card indicating their deficiency status and containing a list of drugs to be avoided or dosed carefully by qualified medical staff. Specific G6PD deficiency-sensitive training for doctors in all malaria-affected districts of Tajikistan should be offered to raise awareness about this issue. In a first phase, this might focus on districts with a higher proportion of ethnic Uzbeks.

## Authors' contributions

CER planned and led the survey on G6PD deficiency and was the main author of the article. AJM collected data, analysed the influence of major environmental factors regarding the distribution of malaria, and developed the malaria risk map. KS took part in the planning of activities, assisted with obtaining agreement from the Ministry of Health, and informed local public authorities and hospital directors about the G6PD deficiency survey. KW and DM conceived the study, assisted AJM and CER in designing and discussing their research, and contributed to the elaboration of the manuscript.
